# Carbonic Anhydrase I and II as Biomarkers and Therapeutic Targets in Human Disease: From Physiology to Clinical Translation

**DOI:** 10.3390/ijms27146375

**Published:** 2026-07-17

**Authors:** Ayşegül Sümer, Sera Şahin, Ahmet Menteşe

**Affiliations:** 1 Department of Medical Biochemistry, Faculty of Medicine, Recep Tayyip Erdoğan University, Rize 53100, Türkiye; 2 Department of Medical Biochemistry, Graduate School of Health Sciences, Karadeniz Technical University, Trabzon 61080, Türkiye; sera07809@gmail.com; 3 Department of Medical Biochemistry, Faculty of Medicine, Karadeniz Technical University, Trabzon 61080, Türkiye; amentese28@ktu.edu.tr

**Keywords:** carbonic anhydrase I, carbonic anhydrase II, biomarkers, therapeutic targets, carbonic anhydrase inhibitors, glaucoma, obstructive sleep apnea, atherosclerosis, anemia, neurodegeneration

## Abstract

Carbonic anhydrases (CAs) are zinc-containing metalloenzymes that catalyze the reversible conversion of carbon dioxide and water into bicarbonate and protons, contributing to acid–base balance, pH regulation, and ion transport. Among human cytosolic isoforms, carbonic anhydrase I (CA I) and carbonic anhydrase II (CA II) are abundant and clinically relevant, yet their distinct roles are often obscured within broader discussions of the CA family. This narrative review evaluates CA I and CA II as biomarkers and therapeutic targets in glaucoma, atherosclerosis and vascular calcification, anemia, epilepsy, Alzheimer’s disease, obstructive sleep apnea, obesity-related metabolic dysfunction, and selected cancers. CA II emerges as the more established pharmacological target, particularly in glaucoma, with acetazolamide and sultiame showing therapeutic potential in obstructive sleep apnea and possible contributions to epilepsy and neurodegeneration through pH regulation, bicarbonate-dependent signaling, and mitochondrial function. CA I instead appears more valuable as a disease-associated biomarker, especially in disorders involving erythrocyte turnover, inflammation, anemia, and malignancy, though circulating CA I may be confounded by hemolysis and altered erythrocyte dynamics. Clinical translation requires isoform-selective modulators, tissue-targeted delivery, standardized biomarker assays, and mechanistic models distinguishing primary CA involvement from secondary disease-related changes.

## 1. Introduction

Carbonic anhydrases (CAs; EC 4.2.1.1) are zinc-containing metalloenzymes that catalyze the reversible hydration of carbon dioxide (CO_2_) to bicarbonate (HCO_3_^−^) and protons (H^+^). Through this reaction, they play a central role in acid–base homeostasis, respiratory gas transport, ion exchange, and epithelial fluid secretion [1,2,3,4]. Since their first purification from erythrocytes of several mammalian species by Meldrum and Roughton in 1933, CAs have been recognized as key enzymes linking cellular physiology, metabolism, and disease [5]. Eight genetically distinct CA families have been identified across living organisms. In humans, however, only the α-class carbonic anhydrases are expressed, and this class accounts for all physiologically and pharmacologically relevant CA activity in human tissues [1,3,4].

Sixteen α-CA isoforms have been described in humans, 15 of which are expressed; CA XV is not present in human tissues [1,6]. Although these isoforms share a conserved Zn^2+^-containing active site coordinated by three histidine residues, they differ substantially in their subcellular localization, tissue distribution, catalytic efficiency, and pharmacological sensitivity [2,4,7,8]. According to localization, human CAs are generally classified as cytosolic isoforms (CA I, II, III, VII, XIII), mitochondrial isoforms (CA VA and VB), membrane-associated isoforms (CA IV, IX, XII, XIV), and the secreted isoform CA VI [1,3,6]. In addition, CA VIII, CA X, and CA XI lack catalytic activity because they do not contain the zinc-coordinating histidine residues required for enzymatic function; these proteins are therefore classified as carbonic anhydrase-related proteins (CARPs) [2,6]. This compartmentalized distribution allows different CA isoforms to regulate local pH environments and support tissue-specific physiological processes.

Among the cytosolic isoforms, CA I and CA II are particularly important because of their abundance, physiological roles, and clinical relevance. CA I is the second most abundant protein in human erythrocytes after hemoglobin. Although its catalytic activity is only about 15% of that of CA II, CA I is present in erythrocytes at approximately five- to six-fold higher concentrations than CA II [6,9,10]. This high abundance makes CA I an important contributor to CO_2_ transport and intracellular buffering in erythrocytes. CA II, in contrast, is one of the most catalytically efficient enzymes known and has a much broader tissue distribution. It is expressed in erythrocytes, renal tubular cells, ocular tissues, brain oligodendrocytes, choroid plexus epithelium, osteoclasts, gastric mucosa, skeletal muscle, and hepatocytes [2,9,11]. This wide expression profile explains its involvement in renal acid secretion and bicarbonate reabsorption, aqueous humor formation, cerebrospinal fluid dynamics, bone resorption, and neuronal pH regulation [6,12,13].

Over the past two decades, increasing evidence has linked altered CA I and CA II expression or activity to several human diseases, including vascular calcification and atherosclerosis, epilepsy, Alzheimer’s disease and other neurodegenerative conditions, anemia, glaucoma, obstructive sleep apnea, and obesity-related metabolic dysfunction [9,11,14,15,16,17]. From a pharmacological perspective, CA II has the stronger clinical foundation. It is the main target of established carbonic anhydrase inhibitors such as acetazolamide (AZM), methazolamide (MTZ), dorzolamide, and brinzolamide, which are used in conditions including glaucoma, edema, epilepsy, altitude sickness, and idiopathic intracranial hypertension [6,17,18]. More recently, sultiame, another carbonic anhydrase inhibitor, has shown efficacy in obstructive sleep apnea in randomized clinical trials [19,20]. CA I, by contrast, has not yet been directly targeted in routine clinical practice. Instead, it is increasingly being investigated as a disease-associated biomarker in hematological, inflammatory, vascular, and selected oncological settings [9,15,21,22].

A major difficulty in interpreting the CA literature is the tendency to generalize findings across isoforms. This is particularly problematic because different CA isoforms often have distinct biological and clinical roles. In cancer biology, for example, the hypoxia-inducible membrane isoforms CA IX and CA XII have much clearer mechanistic links to extracellular acidification, tumor invasion, and therapy resistance than CA I or CA II [23,24,25]. Similarly, in obesity and metabolic disease, the mitochondrial isoforms CA VA and CA VB are more directly involved in bicarbonate-dependent lipogenesis and gluconeogenesis than the cytosolic isoforms [26,27]. For this reason, any meaningful evaluation of CA I and CA II must distinguish isoform-specific evidence from broader CA family effects. Although several reviews have discussed the CA family as a whole or focused on carbonic anhydrase inhibitor development [6,17,18,28], a focused and critical synthesis of CA I and CA II remain needed. Their disease associations should be evaluated in terms of evidence strength, isoform specificity, and their relative potential as biomarkers or therapeutic targets.

These existing reviews differ from the present work in scope and organizing principle rather than topic. Mishra et al. and the more recent polypharmacology review by Supuran provide comprehensive medicinal-chemistry surveys of CA inhibitor development across the full 15-isoform family, organized by chemical inhibitor class rather than by disease or isoform-specific evidence strength [6,17]. Supuran’s earlier analysis catalogues the known mechanisms of CA inhibition mechanistically, independent of disease context [12]. Zamanova et al. review CA family members broadly as disease markers without separating CA I from CA II on a disease-by-disease basis [9]. Poggetti et al. focus specifically on CA activators for neurodegeneration, a narrower pharmacological and disease scope than considered here [10]. None of these reviews organizes evidence explicitly by isoform (CA I versus CA II), assigns an explicit qualitative evidence grade to each disease association, or proposes a translational framework distinguishing biomarker candidacy from pharmacological actionability; this isoform-specific, evidence-graded framing across eight disease contexts is the specific contribution of the present review.

This narrative review addresses this gap by offering an isoform-specific perspective on CA I and CA II in human disease. Its main contribution is to frame these two cytosolic enzymes differently: CA I is discussed primarily as a context-dependent disease biomarker, whereas CA II is considered a more established and clinically actionable pharmacological target. Rather than treating CA I and CA II as interchangeable members of the broader CA family, this review evaluates their disease associations separately, with attention to the strength of evidence, biological plausibility, and translational relevance. It also considers whether reported changes in CA I or CA II are likely to represent causal mechanisms, compensatory responses, or secondary effects of processes such as inflammation, erythrocyte turnover, and oxidative stress. The review focuses on glaucoma, atherosclerosis and vascular calcification, anemia, epilepsy, Alzheimer’s disease, obstructive sleep apnea, obesity-related metabolic dysfunction, and the potential value of CA I as a cancer biomarker. The mechanisms underlying CA inhibition and activation are also discussed, particularly in relation to their clinical relevance and the current challenges limiting their translation into practice.

## 2. Search Strategy and Selection Criteria

This article was designed as a narrative review because the available literature on CA I and CA II include heterogeneous evidence from biochemical studies, structural analyses, pharmacological investigations, experimental disease models, clinical observations, and biomarker studies. Therefore, a pooled quantitative synthesis was not considered appropriate.

A structured literature search was performed using PubMed, Scopus, Web of Science, and Google Scholar. The following keywords and their combinations were used: “carbonic anhydrase,” “carbonic anhydrase I,” “CA I,” “carbonic anhydrase II,” “CA II,” “carbonic anhydrase inhibitor,” “carbonic anhydrase activator,” “biomarker,” “therapeutic target,” “isoform selectivity,” “pharmacological modulation,” and “clinical translation.” These terms were combined with disease-specific keywords, including “glaucoma,” “atherosclerosis,” “vascular calcification,” “anemia,” “epilepsy,” “Alzheimer’s disease,” “obstructive sleep apnea,” “obesity,” “metabolic dysfunction,” and “cancer.”

Priority was given to peer-reviewed original articles, clinical studies, randomized or interventional trials, mechanistic experimental studies, structural biology studies, pharmacological investigations, and relevant review articles. Recent publications from the last 10–15 years were prioritized, while older seminal studies were included when necessary to provide historical, biochemical, or mechanistic context. Studies were considered eligible for discussion when they provided direct or clearly interpretable evidence related to CA I or CA II expression, activity, disease association, biomarker potential, pharmacological modulation, or therapeutic relevance.

Articles were excluded from detailed discussion if they addressed the carbonic anhydrase family only in general terms without specific information on CA I or CA II, if they focused exclusively on other CA isoforms without relevance to CA I/II comparison, if methodological details were insufficient, or if only abstracts were available. Because this review was not designed as a systematic review or meta-analysis, no formal risk-of-bias assessment or pooled effect estimation was performed. The selected literature was synthesized narratively, with emphasis on isoform specificity, strength of evidence, biological plausibility, and translational relevance.

## 3. Biological Basis of CA I and CA II

### 3.1. Structural Features and Catalytic Mechanism

Human CA I and CA II belong to the α-carbonic anhydrase class and share a highly conserved three-dimensional structure. Both enzymes contain a central ten-stranded β-sheet surrounded by α-helices and loop regions [2,7,8]. Their catalytic active site lies at the base of a conical cavity, approximately 15 Å deep. Within this cavity, a Zn^2+^ ion is coordinated in a tetrahedral geometry by three histidine residues, His94, His96, and His119, together with a fourth ligand that is either a water molecule or a hydroxide ion, depending on the protonation state of the enzyme [2,4,7]. This Zn^2+^-binding arrangement is conserved among catalytically active human CA isoforms and is essential for enzyme activity [8,12].

The active-site cavity has an amphiphilic character. Its hydrophobic region, formed by residues such as Val121, Phe131, Val135, Leu141, Val143, Leu198, and Trp209, helps position CO_2_ for catalysis. In contrast, the hydrophilic region, which includes Asn62, His64, Asn67, Gln92, Thr199, and Thr200, supports hydrogen bonding and proton transfer [2,7,29]. Two residues are particularly important in this process: Thr199 and Glu106. By forming hydrogen bonds with the Zn^2+^-bound water or hydroxide, these residues increase the nucleophilicity of the metal-coordinated species and help orient the substrate within the active site [4,12,29].

The catalytic cycle of CA I and CA II follows a two-step ping-pong mechanism. In the first step, the Zn^2+^-bound hydroxide ion acts as a nucleophile and attacks CO_2_ positioned in the hydrophobic pocket. This reaction produces bicarbonate (HCO_3_^−^), which is then displaced from the active site by a water molecule. In the second step, which is rate-limiting under physiological conditions, the Zn^2+^-bound water molecule is deprotonated to regenerate the active Zn^2+^-hydroxide form of the enzyme [4,8,12]. This proton-transfer step is mediated mainly by His64, which acts as an intramolecular proton shuttle. His64 alternates between an “in” conformation, directed toward the Zn^2+^ ion, and an “out” conformation, directed toward the solvent [7,29]. Because the pKa of His64 is close to physiological pH, it is well suited for this proton-shuttling role. Residues Asn62, Asn67, and Gln92 further stabilize the hydrogen-bond network around His64 and contribute to efficient proton transfer [2,4,29].

Despite sharing the same basic catalytic framework, CA I and CA II differ in ways that strongly influence their catalytic efficiency. CA I was first sequenced between 1972 and 1974 and consists of 260 amino acids, with a molecular weight of approximately 30 kDa [29,30]. CA II, first characterized crystallographically by Liljas and colleagues in 1972, contains 259 amino acids and has a similar molecular weight [7,8]. Structurally, the two enzymes are closely related, but their catalytic performance differs markedly. CA II has a catalytic turnover rate (kcat) of approximately 10^6^ s^−1^, making it one of the most efficient enzymes known, whereas CA I operates at roughly 15% of this rate [6,9]. This difference is mainly related to the proton-transfer step. CA II has a more favorable active-site geometry and a better-positioned His64 proton shuttle, while CA I has a less efficient proton-transfer pathway because of subtle differences in the hydrogen-bonding network around the active site [2,7,12].

Thus, although CA I and CA II catalyze the same reaction, their biological contributions are not determined by catalytic rate alone. Their roles in vivo also depend on their relative abundance, tissue distribution, and the physiological context in which each isoform is expressed.

### 3.2. Tissue Distribution and Physiological Roles

CA I is expressed predominantly in erythrocytes, where it is the second most abundant protein after hemoglobin [6,9]. Although its catalytic efficiency is lower than that of CA II, CA I is present in erythrocytes at five- to six-fold higher concentrations, making its overall contribution to intracellular CO_2_ hydration and pH buffering physiologically important, particularly under conditions of high CO_2_ flux [6,10]. Beyond erythrocytes, CA I expression has also been reported in the colon, kidney, and, to a lesser extent, motor neurons of the human spinal cord, suggesting that its functions are not limited to erythrocyte physiology [1,9]. In the kidney, CA I participates in tubular acid–base handling by contributing to H^+^ secretion and HCO_3_^−^ reabsorption, although this role is generally considered secondary to that of CA II in the same tissue [6,12]. Circulating CA I levels are normally low, but they can increase markedly in certain pathological conditions. For this reason, CA I is currently viewed more as a candidate disease-associated biomarker than as a primary pharmacological target [9,21].

CA II has the broadest tissue distribution among the cytosolic CA isoforms and supports essential functions in several organ systems. It is highly expressed in erythrocytes, renal proximal and distal tubular cells, ocular tissues including the retina, lens, and ciliary body, brain oligodendrocytes, choroid plexus epithelium, osteoclasts, gastric mucosa, skeletal muscle, testis, pancreas, lungs, salivary glands, and hepatocytes [2,9,11]. This wide distribution reflects the need for rapid CO_2_–HCO_3_^−^ interconversion in many physiological compartments. In the kidney, CA II is the dominant isoform involved in bicarbonate reabsorption and urinary acidification, both of which are essential for systemic acid–base balance [6,13]. In the eye, CA II contributes to aqueous humor production in the ciliary body, explaining its established role as a pharmacological target in glaucoma treatment [13,16]. In bone, CA II is required for osteoclast-mediated bone resorption by helping generate the acidic microenvironment needed for mineral dissolution [6,13]. In the central nervous system, it helps maintain pH homeostasis in oligodendrocytes and contributes to cerebrospinal fluid regulation through the choroid plexus [1,11]. CA II also supports cellular metabolism by working alongside mitochondrial and membrane-associated CA isoforms to provide HCO_3_^−^ for biosynthetic carboxylation reactions involved in gluconeogenesis and lipogenesis [6,12].

### 3.3. Key Differences Between CA I and CA II: Catalytic Efficiency, Tissue Abundance, and Clinical Relevance

Although CA I and CA II are structurally similar, their biological and clinical roles differ in important ways. CA II is the more catalytically efficient isoform and has a broader tissue distribution, which explains its more direct involvement in organ-level physiology and its established role as a target of clinically used carbonic anhydrase inhibitors [6,12,17]. CA I, in contrast, is quantitatively dominant in erythrocytes but has a narrower tissue distribution and a lower catalytic rate. Its main physiological contribution is therefore largely linked to the erythrocyte compartment, where its high abundance partly compensates for its lower enzymatic efficiency [6,9,10]. The clinical consequences of isoform deficiency also reflect this difference. CA II deficiency causes a severe multisystem disorder characterized by renal tubular acidosis, osteopetrosis, and cerebral calcification, whereas isolated CA I deficiency is generally mild or subclinical [13].

These differences are also important from a translational perspective. Because CA I is highly concentrated in erythrocytes, it can be released into circulation during erythrocyte lysis, hemolysis, or inflammation-associated changes in erythropoiesis. As a result, plasma or serum CA I levels may reflect pathological processes involving erythrocyte turnover, oxidative stress, or vascular inflammation [9,22]. This has made CA I a candidate biomarker in anemia, atherosclerosis, and selected malignancies. CA II, by contrast, is more directly involved in tissue-specific processes that can be targeted pharmacologically, including aqueous humor secretion, renal acid–base handling, and neuronal pH regulation [6,13,17]. The established clinical use of CA II-targeting inhibitors such as acetazolamide, dorzolamide, and brinzolamide supports its status as a validated therapeutic target, while emerging evidence suggests that CA II-directed pharmacology may also be relevant in obstructive sleep apnea and neurodegenerative disease [17,19,20].

**Figure 1:**
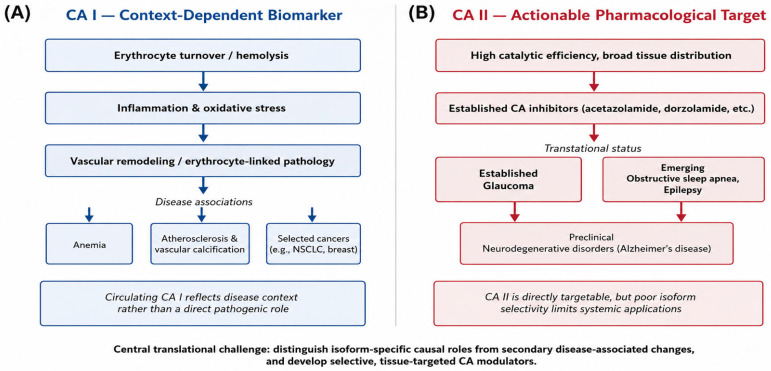
Isoform-specific translational framework for CA I and CA II in human disease. CA I and CA II share the same catalytic reaction but differ substantially in tissue distribution, catalytic efficiency, disease relevance, and translational potential. (**A**) CA I is highly abundant in erythrocytes and is mainly positioned as a context-dependent biomarker candidate in conditions associated with erythrocyte turnover, hemolysis, inflammation, oxidative stress, vascular remodeling, anemia, atherosclerosis, and selected cancers. (**B**) CA II has higher catalytic efficiency and broader tissue distribution, making it a more established and clinically actionable pharmacological target, particularly in glaucoma, and an emerging or preclinical target in obstructive sleep apnea, epilepsy, and neurodegenerative disorders. The central challenge for clinical translation, common to both isoforms, is to distinguish isoform-specific causal roles from secondary disease-associated changes and to develop selective, tissue-targeted CA modulators.

Together, these observations provide the framework used throughout this review: CA I is considered primarily as a disease-context-dependent biomarker, whereas CA II is evaluated as a pharmacologically actionable enzyme. The principal biological and translational differences between CA I and CA II are summarized in Table 1 and the isoform-specific translational framework proposed in this review is illustrated in Figure 1, in which CA I is positioned mainly as a context-dependent biomarker and CA II as a clinically actionable pharmacological target.

## 4. Pharmacological Modulation of CA I and CA II

Carbonic anhydrases are important pharmacological targets, and their enzymatic activity has been modulated through both inhibition and activation for many years in clinical and experimental settings. Among this enzyme family, CA I and CA II are particularly relevant because they are the main cytosolic isoforms found in erythrocytes and are also expressed in many metabolically active tissues. As a result, drugs developed to target carbonic anhydrases often affect these two isoforms, even when they are not the primary intended targets. Therefore, understanding how CA I and CA II are inhibited or activated is essential for explaining both the therapeutic actions and possible adverse effects of CA-directed compounds, as well as for assessing the isoform selectivity of current and emerging drugs [6,12,17].

### 4.1. Mechanisms of Carbonic Anhydrase Inhibition

To date, carbonic anhydrase inhibitors (CAIs) have been grouped into five mechanistically distinct classes, based on how they interact with the enzyme active site [12,34]. Although these classes may all have therapeutic relevance, they differ considerably in their isoform selectivity, pharmacokinetic properties, and potential adverse effects.

#### 4.1.1. Zinc-Binding Inhibitors

Zinc-binding inhibitors are the classical and most widely studied group of carbonic anhydrase inhibitors. These compounds contain a zinc-binding group (ZBG), which directly coordinates the catalytic Zn^2+^ ion in the enzyme active site. By doing so, they replace the Zn^2+^-bound water or hydroxide molecule that is normally required for CO_2_ hydration [7,12,34]. Primary sulfonamides (R–SO_2_NH_2_) are the prototypical ZBGs and have formed the main chemical basis of CA inhibitor development for more than seven decades. In addition to sulfonamides, several other zinc-binding scaffolds have been described, including sulfamates, sulfamides, dithiocarbamates, monothiocarbamates, hydroxamates, and carboxylates [12,34,35].

The binding mode of these inhibitors may vary according to the chemical structure of the compound and the microenvironment of the active site. In general, ZBG-containing inhibitors coordinate the catalytic zinc in either tetrahedral or trigonal bipyramidal geometries. Their binding is further stabilized by hydrogen-bonding interactions with key residues such as Thr199 and Glu106, which contribute to both inhibitory potency and isoform selectivity [7,12,29].

Several sulfonamide-based CAIs are already used clinically. Acetazolamide (AZM) and methazolamide (MTZ) are used in conditions such as glaucoma, altitude sickness, idiopathic intracranial hypertension, and epilepsy. Dorzolamide and brinzolamide are used as topical anti-glaucoma agents, whereas topiramate and zonisamide are primarily antiepileptic drugs that also show secondary CA-inhibitory activity [6,17,18]. In addition, some diuretics, including hydrochlorothiazide, furosemide, and bumetanide, can inhibit CA I and CA II in vitro, suggesting that off-target CA inhibition may contribute to some of their pharmacological actions [35].

However, the major limitation of zinc-binding inhibitors is their relatively poor isoform selectivity. Since the Zn^2+^-coordination region is highly conserved among human CA isoforms, classical sulfonamides often inhibit several isoenzymes at the same time. This broad inhibition profile is one of the main reasons for systemic adverse effects such as paresthesia, fatigue, metabolic acidosis, nephrolithiasis, and gastrointestinal discomfort [6,17,18].

#### 4.1.2. Inhibitors Targeting the Zn^2+^-Coordinated Water/Hydroxide Ion

A second group of carbonic anhydrase inhibitors acts through a different mechanism. Instead of directly coordinating the catalytic Zn^2+^ ion, these compounds interact with the Zn^2+^-bound water or hydroxide molecule in the active site through hydrogen bonding. Phenols were the first inhibitors shown to act in this way, and they were later followed by polyamines, including spermine and spermidine derivatives [12,34].

These inhibitors generally contain an anchoring group that enables interaction with the metal-coordinated water species. In phenols and polyphenols, this role is usually played by a hydroxyl group, whereas in polyamines it is provided by a protonated amine. The remaining part of the molecule, which may be aromatic, aliphatic, or heterocyclic, contributes additional hydrophobic and van der Waals interactions within the active-site cavity [7,12,34].

Other compounds reported to act through this mechanism include iodide ion, hydrolyzed sulfocoumarins, thiocoumarins, and selected carboxylates. Since the residues surrounding the Zn^2+^-bound water molecule differ to some extent among CA isoforms, this binding mode may provide a useful strategy for developing inhibitors with better isoform selectivity than classical zinc-coordinating sulfonamides [12,34].

#### 4.1.3. Active-Site Entrance Blockers

Coumarins and their derivatives, encompassing sulfocoumarins, thiocoumarins, substituted lactones, thiolactones, and quinolinones, function as inhibitors of carbonic anhydrases. Their inhibitory mechanism involves binding at the entrance of the active-site cavity, distinct from the catalytic metal center itself [12,34,36]. Unlike inhibitors that target zinc, these compounds do not coordinate with Zn^2+^ or interact with the water molecule bound to it. Instead, they obstruct the substrate access channel through steric hindrance, effectively preventing CO_2_ from reaching the catalytic pocket [12,34]. This particular mechanism holds significant interest, as the residues located at the active-site entrance demonstrate less conservation across CA isoforms compared to those within the Zn^2+^-binding core. This structural characteristic offers a foundational basis for achieving enhanced isoform selectivity. Indeed, coumarin-based inhibitors have demonstrated differential selectivity profiles against CA I, CA II, and various other isoforms, underscoring their potential value in the development of isoform-targeted therapeutics [12,34,36].

#### 4.1.4. External-Site Binders and Proton-Transfer Interfering Compounds

A fourth mode of inhibition involves compounds that bind outside the classical catalytic pocket. Unlike zinc-binding inhibitors or compounds that block substrate entry, these inhibitors reduce enzyme activity by interfering with the proton-transfer step of the catalytic cycle. As described in Section 3.1, this proton-transfer step, mediated mainly by His64, is the rate-limiting step in carbonic anhydrase catalysis.

Compounds that stabilize His64 in a non-productive conformation can therefore suppress catalytic turnover without directly occupying the active site. The prototype of this class is 2-(benzenesulfonyl) benzoic acid, which has been reported to trap His64 in its outward-facing conformation through hydrogen-bond interactions. This disrupts the proton shuttle mechanism and consequently reduces enzymatic activity [12,34].

Because this mechanism involves a region outside the highly conserved Zn^2+^-binding core, external-site binders may offer an alternative strategy for developing more selective CA modulators with distinct isoform preferences [12,29,34].

#### 4.1.5. Inhibitors with Incompletely Characterized Mechanisms

A final group includes compounds that have been shown to inhibit human CA isoforms in kinetic assays, but whose exact binding mechanisms are still not fully understood because high-resolution structural data are lacking. This group includes some secondary and tertiary sulfonamides, probenecid amide derivatives, and tyrosine kinase inhibitors such as imatinib and nilotinib. Notably, these compounds have been reported to inhibit CA I and CA II at nanomolar concentrations, even though they do not contain the classical primary sulfonamide zinc-binding group [12,28,34].

At present, it remains unclear whether these inhibitors act through direct interactions within the active site, binding at external sites, conformational changes, or a combination of these mechanisms. Therefore, further crystallographic, biophysical, and computational studies are needed to clarify how they inhibit CA activity and to determine whether they may be useful scaffolds for designing isoform-selective CA inhibitors [12,34].

### 4.2. Carbonic Anhydrase Activation

Carbonic anhydrase activators (CAAs) constitute a pharmacologically distinct group of CA modulators, although they are much less developed clinically than carbonic anhydrase inhibitors. Evidence that carbonic anhydrases can be activated has existed for several decades; however, the molecular basis of this activation became clearer only in the early 1990s, with the use of purified enzyme preparations and stopped-flow kinetic methods [37,38]. Known CAAs include biogenic amines such as histamine, serotonin, and catecholamines, as well as amino acids, oligopeptides, indole-based compounds, and amino alcohol oxime ether derivatives [37,38,39].

The generally accepted mechanism of CA activation involves the formation of an enzyme–substrate–activator ternary complex. In this complex, the activator binds within or near the active-site cavity, but at a site distant from the catalytic Zn^2+^ ion. Unlike CA inhibitors, CAAs do not act by blocking the metal center. Instead, they enhance the proton-transfer step, which is the rate-limiting phase of the catalytic cycle, by providing an additional proton-shuttling route that supports the intrinsic His64-mediated mechanism [37,38].

X-ray crystallographic studies of CA II–activator complexes have shown that activators such as histamine can form hydrogen bonds with Asn62, Asn67, and Gln92 near the entrance of the active site. These interactions reorganize the local hydrogen-bonding network and facilitate proton transfer toward His64 [37,39]. Most known activators bind to a region referred to as “activator binding site A,” which is located on the opposite side of the active site from His64. A notable exception is D-tryptophan, which binds to an alternative external site [37,38].

Preclinical studies have linked CA activation to improved synaptic efficacy, spatial learning, and memory performance in aging rodent models. These findings have increased interest in CAAs as potential therapeutic agents for neurodegenerative and cognitive disorders [10,37]. More recently, amino alcohol oxime ether-based derivatives have been evaluated for their ability to activate brain-associated CA isoforms, including CA II and CA VII, suggesting that isoform-selective activation may be achievable [39]. CA activation has also been proposed to support calcium carbonate formation during bone mineralization, which may be relevant for tissue engineering applications [40,41]. However, the clinical translation of CAAs remains limited by unresolved issues, including isoform specificity, blood–brain barrier penetration, long-term safety, and clinical efficacy. To date, no CAA has entered clinical use [10,37,38].

### 4.3. Isoform Selectivity: Relevance for CA I and CA II

A major and recurring challenge in carbonic anhydrase pharmacology is the limited isoform selectivity of many available inhibitors and activators. Since the catalytic Zn^2+^-binding region is highly conserved among the 15 catalytically active human CA isoforms, classical sulfonamide-based inhibitors often inhibit CA I, CA II, and several other isoforms at the same time [6,12,17].

For CA I and CA II, this lack of selectivity is especially important. Inhibition of CA II may be therapeutically desirable in tissues such as the eye, kidney, or brain, but it is often accompanied by inhibition of CA I in erythrocytes. This may affect CO_2_ transport and acid–base buffering in the circulation [6,18]. In addition, because CA I is highly abundant in erythrocytes, any circulating CA inhibitor is likely to encounter CA I as a major off-target isoform. This helps explain some of the systemic adverse effects of orally administered CAIs such as acetazolamide and methazolamide, including paresthesia, fatigue, and metabolic acidosis. These effects probably reflect combined inhibition of CA II in renal tubules and CA I/CA II in erythrocytes [6,18].

Recent progress in isoform-selective CA modulation has come mainly from non-classical inhibitor classes, particularly compounds that block the active-site entrance or bind to external sites. These inhibitors take advantage of structural differences in less-conserved regions of the active-site cavity [12,34,36]. In parallel, topical delivery strategies, such as ocular formulations of dorzolamide and brinzolamide, provide a practical way to reduce systemic CA I/II inhibition while maintaining effective local CA II inhibition in the ciliary body [6,18].

Future CA-directed therapies will require more selective compounds with clearly defined binding mechanisms, pharmacokinetic properties, and tissue distribution profiles. Such advances will be essential for achieving targeted therapeutic effects while minimizing unwanted inhibition of other CA isoforms [12,17,34]. Representative CA inhibitors and activators relevant to CA I and CA II, together with their clinical or translational contexts, are summarized in Table 2.

## 5. CA I and CA II in Human Disease

The following sections evaluate the evidence linking CA I and CA II to specific human diseases. For each condition, evidence is assessed with explicit attention to isoform specificity, evidence strength, and whether CA I/II alterations represent primary pathogenic mechanisms or secondary responses to inflammation, erythrocyte turnover, oxidative stress, or other disease processes. The diseases are presented in order of decreasing strength of CA II evidence, beginning with glaucoma where the therapeutic role of CA II is most firmly established and proceeding to conditions where the evidence remains more associative or preliminary.

### 5.1. Glaucoma

Glaucoma is a progressive optic neuropathy that is commonly associated with elevated intraocular pressure (IOP). This increase in pressure usually reflects an imbalance between aqueous humor production and outflow. In the ciliary body epithelium, CA II is the main cytosolic isoform responsible for bicarbonate production. Bicarbonate formation promotes sodium and fluid movement into the posterior chamber, thereby contributing to aqueous humor generation [6,13]. Although membrane-associated isoforms such as CA IV and CA XII also participate in ocular fluid regulation, CA II remains the principal pharmacological target for lowering IOP [31,32].

Systemic carbonic anhydrase inhibitors, including acetazolamide, methazolamide, dichlorphenamide, and ethoxzolamide, were among the first drugs used to reduce IOP by decreasing aqueous humor formation. However, their long-term use is limited by systemic adverse effects. These effects are largely related to simultaneous inhibition of CA II in renal tubules and CA I/CA II in erythrocytes, leading to fatigue, paresthesia, metabolic acidosis, electrolyte disturbances, and nephrolithiasis [6,17,18]. This limitation led to the development of topical CA inhibitors. Dorzolamide and brinzolamide penetrate the cornea and act locally in the ciliary processes, where they reduce bicarbonate-dependent aqueous humor secretion while producing much lower systemic exposure [31,32]. As a result, topical CA inhibitors generally have a better systemic safety profile, although local adverse effects such as ocular irritation, allergic conjunctivitis, corneal edema, and metallic or bitter taste may still occur [32].

In clinical practice, topical CA inhibitors are mainly used as adjunctive therapy when IOP is not adequately controlled with prostaglandin analogues, β-blockers, or α_2_-adrenergic agonists. They are also available in fixed-combination formulations, which can improve treatment adherence [31,32]. Beyond conventional pharmacological inhibition, Jiang et al. showed that CRISPR-Cas9-mediated deletion of the CA2 gene in the ciliary body produced sustained IOP reduction after a single intravitreal injection in experimental glaucoma models [31]. This finding suggests that targeted gene-based suppression of CA II may represent a future therapeutic strategy. However, before clinical translation, this approach will require careful evaluation of long-term safety, off-target effects, reversibility, and preservation of physiological CA II functions in extraocular tissues.

CA I does not have a major direct role in aqueous humor dynamics. Its relevance in glaucoma is therefore mostly indirect. Systemic CA I inhibition may contribute to some adverse effects of oral CAIs, and increased CA expression, including CA I, has been proposed as a possible shared pathological feature linking glaucoma and atherosclerosis in patients with comorbid disease [51]. Overall, among the conditions discussed in this review, the pharmacological relevance of CA II is most strongly supported in glaucoma. Its central role in aqueous humor secretion has been therapeutically exploited for decades, and the shift from systemic to topical inhibition clearly illustrates how drug delivery strategy can convert a broadly acting intervention into a more targeted and better tolerated treatment.

### 5.2. Atherosclerosis and Vascular Calcification

Vascular calcification (VC) refers to the pathological deposition of calcium phosphate minerals within the arterial wall. It contributes to arterial stiffness, plaque instability, and increased cardiovascular risk. In atherosclerosis (AS), VC is generally classified into two main forms: intimal calcification, which is associated with lipid accumulation, macrophage infiltration, and chronic inflammation, and medial calcification, which is mainly linked to vascular smooth muscle cell (VSMC) dysfunction and elastic fiber degradation [52,53]. A key process in VC is the osteogenic transdifferentiation of VSMCs, during which vascular cells acquire osteoblast-like properties and increase the expression of markers such as Runx2, BMP2, and alkaline phosphatase (ALP) [15,52].

Experimental findings suggest that both CA I and CA II may contribute to the development of VC and AS. Song et al. showed that M1-polarized macrophages secrete TNF-α, which increases CA I and CA II expression in VSMCs and promotes the expression of calcification-related genes, including Runx2, BMP2, and ALP. Inhibition of CA I and CA II by siRNA reduced β-glycerophosphate-induced calcification, whereas TNF-α exposure restored CA expression and calcification [54]. These results suggest that inflammation-induced CA expressions may provide a mechanistic link between macrophage activation and vascular mineralization.

CA I has a more direct role in AS progression. Zong et al. used a CRISPR/Cas9-generated ApoE^−^/^−^ CA I-overexpressing knock-in mouse model fed a high-fat diet and showed that CA I overexpression aggravated AS. This was associated with increased M1-type macrophage accumulation, aortic wall thickening, and plaque burden, while concurrent treatment with methazolamide (MTZ) partially reduced these changes [44]. Similarly, Yuan et al. reported that CA I expression correlated with AS severity in human tissues and experimental models. They also showed that acetazolamide (AZM) and MTZ suppressed CA I expression together with osteogenic markers and pro-inflammatory cytokines, including IL-6, IFN-γ, GM-CSF, and TNF-α [15]. These findings support the view that CA I may act both as a mediator of calcification-related inflammation and as a potential pharmacological target in AS, although the available evidence remains largely preclinical.

For CA II, the evidence is more indirect. Oksala et al. reported increased CA II expression in osteoclast-like cells within advanced human atherosclerotic plaques, while Ayari and Bricca identified CA II overexpression in human carotid plaques by microarray analysis [55,56]. In addition, Argan et al. showed that several commonly prescribed cardiovascular drugs can inhibit CA I and CA II in vitro, raising the possibility that ancillary CA inhibition may contribute to some of their effects in patients with AS [35]. Song et al. also reported a clinical association between glaucoma and AS-related parameters in patients receiving MTZ, suggesting that the two conditions may share CA-mediated pathways; however, this observation remains associative [51].

Taken together, the current atherosclerosis literature supports a more clearly defined mechanistic role for CA I than for CA II. Experimental models consistently link CA I to inflammation-driven osteogenic reprogramming of VSMCs and macrophage-mediated plaque progression. In contrast, changes in CA II expression in advanced plaques may reflect local adaptive responses rather than a primary disease-driving mechanism. Whether CA I or CA II can serve as clinically useful biomarkers or therapeutic targets in atherosclerotic cardiovascular disease will require prospective human studies and more isoform-selective mechanistic tools.

### 5.3. Anemia

Because CA I and CA II together make up a substantial proportion of erythrocyte proteins, changes in their expression or activity during anemia may provide useful information about erythrocyte pathophysiology. These isoenzymes contribute to CO_2_ transport, intracellular pH buffering, and acid–base regulation. Therefore, their levels may be affected in conditions involving erythrocyte dysfunction, increased erythrocyte turnover, oxidative damage, or immune-mediated destruction [6,9,22].

Early studies reported altered erythrocyte CA I levels in different types of anemia. Mondrup and Anker described changes in CA isoenzyme B, later known as CA I, in patients with various anemia subtypes [57]. Kuo et al. examined erythrocyte CA isoenzymes in 118 patients with aplastic anemia, autoimmune hemolytic anemia, iron deficiency anemia, or β-thalassemia. They found that total CA activity and CA II concentration were significantly increased in several anemia groups compared with healthy controls. In contrast, CA I concentration was significantly decreased in autoimmune hemolytic anemia (*p* < 0.01). Based on these findings, the authors suggested that reduced CA I may help distinguish autoimmune hemolytic anemia from other anemia subtypes [22].

In glucose-6-phosphate dehydrogenase (G6PD) deficiency, Chiang et al. reported significantly lower erythrocyte CA I expression in patients with hemolytic anemia compared with controls, whereas CA II expression appeared to increase, possibly as a compensatory response [58]. However, the mechanism underlying this opposite CA I/CA II response in G6PD-related hemolysis remains unclear. In SOD1-knockout mice, increased erythrocyte oxidative stress caused anemia and induced autoantibody production against CA II, suggesting that oxidative modification of erythrocyte CA II may contribute to autoimmune responses [59]. Similarly, Menteşe et al. detected anti-CA antibodies in patients with iron deficiency anemia, further supporting a possible relationship between erythrocyte CA antigenicity and immune-mediated anemia [60].

Overall, erythrocyte CA isoenzyme patterns in anemia appear to be subtype dependent. Rather than representing a single common disease signature, they seem to reflect the underlying pathophysiological process. Reduced CA I in autoimmune hemolytic anemia and the divergent CA I/CA II response observed in G6PD deficiency may have value as complementary biochemical indicators. However, their clinical usefulness still needs to be confirmed in prospective studies using well-defined patient cohorts and standardized assay methods. At present, neither CA I nor CA II can be recommended as a routine diagnostic biomarker for any specific anemia subtype.

### 5.4. Epilepsy

Neuronal excitability is closely related to local pH regulation and CO_2_/HCO_3_^−^ balance in the central nervous system (CNS). In general, extracellular alkalosis increases neuronal firing, whereas acidosis suppresses neuronal activity. Since carbonic anhydrase isoforms regulate the CO_2_–HCO_3_^−^ buffering system in the CNS, they may influence seizure susceptibility by affecting pH, proton concentration, and ion flux [6,46].

Among the CA isoforms, CA II and CA VII are particularly relevant to epilepsy. Both isoforms can enhance bicarbonate-dependent excitatory GABAergic responses during intense neuronal activity. Under prolonged GABA-A receptor activation, intracellular CA activity sustains HCO_3_^−^ efflux, which may depolarize postsynaptic neurons and shift GABAergic signaling from inhibitory to excitatory. Using CA VII and CA II knockout mouse models, Ruusuvuori et al. showed that these cytosolic isoforms amplify bicarbonate-dependent excitatory GABAergic responses and contribute to febrile seizure susceptibility during early postnatal development [46]. CA I has also been linked to epilepsy more indirectly. Luo et al. reported that elevated zinc levels, which can influence zinc-containing enzymes such as CAs, were associated with epilepsy risk and gray matter volume changes in zinc-rich brain regions. In this context, CA I was proposed to contribute to blood flow regulation in the caudate nucleus [61].

Several clinically used antiepileptic drugs (AEDs) also show CA-inhibitory activity. Acetazolamide (AZM) and methazolamide (MTZ) have established anticonvulsant effects. Topiramate and zonisamide primarily act through voltage-gated ion channels and glutamatergic/GABAergic pathways, but they also inhibit CA II, suggesting that pH modulation may contribute to part of their antiseizure activity [6,17,46,62]. Magheru et al. reported that chronic administration of several AEDs, including carbamazepine, phenytoin, valproate, primidone, clonazepam, and ethosuximide, progressively reduced erythrocyte CA II activity in epileptic patients in vivo. Carbamazepine produced the greatest CA II inhibition, reported as 52.75%, and this was associated with a 72% reduction in seizure frequency in that group [63]. These findings suggest that CA II inhibition may contribute to antiseizure efficacy, although it is unlikely to be the only mechanism for most AEDs.

Overall, CA II is the main CA isoform of pharmacological interest in epilepsy because of its expression in CNS glial cells and its role in neuronal pH buffering. CA I appears to have a more secondary role, probably related mainly to systemic acid–base regulation rather than direct neuronal effects. Future studies using isoform-selective CA modulators are needed to clarify the separate contributions of CA II and CA VII to seizure generation and control, and to determine whether selective CA II targeting could improve the therapeutic index of anticonvulsant therapy.

### 5.5. Alzheimer’s Disease

A growing body of evidence links CA I and CA II to Alzheimer’s disease (AD), mainly through pathways involving mitochondrial dysfunction, oxidative stress, amyloid-β (Aβ) toxicity, and impaired neuronal pH regulation. Among these isoforms, CA II has received greater attention because of its high catalytic activity, expression in the central nervous system, and susceptibility to oxidative modification under neurodegenerative conditions [10,64].

In a proteomic study using the Purkinje cell degeneration mouse model, increased CA II levels were detected in cerebellar and retinal mitochondria. This suggests that mitochondria-associated CA II may increase as part of an aging- or stress-related adaptive response [64]. Clinically, plasma CA II levels have been reported to be significantly higher in AD patients than in cognitively normal controls, while patients with mild cognitive impairment showed intermediate levels. A positive correlation between CA II levels and age was also most evident in the AD group [65]. In addition, CA II has been detected in association with amyloid plaques in AD brain tissue, suggesting that it may contribute to plaque-related pH dysregulation or local inflammatory responses [65].

The possible neuroprotective role of CA inhibition has been investigated in several experimental AD models. Methazolamide (MTZ) reduced Aβ-induced mitochondrial dysfunction, decreased ROS production, suppressed caspase activation, and protected neuronal and glial cells both in vitro and in a transgenic AD mouse model [45]. In the TgSwDI model, both MTZ and acetazolamide (AZM) reduced memory impairment and amyloid burden [45]. These findings suggest that early CA II-directed inhibition may help limit mitochondrial injury in AD, although this has not yet been confirmed clinically.

At the same time, CA activation has also been proposed as a potential therapeutic strategy in AD. This idea is based on evidence that CA I and CA II undergo oxidative modification in the frontal cortex and hippocampus of AD brains, which may reduce their catalytic activity and impair pH homeostasis, protein quality control, and synaptic function [10]. Administration of the CAA D-phenylalanine improved synaptic efficacy, spatial learning, and memory in aging rodents [48]. Another study showed that pharmacological modulation of CA activity in the hippocampus and prefrontal cortex produced region-dependent effects on social recognition memory in rats [49]. A recent critical appraisal also identified both CA inhibitors and CA activators as emerging pharmacological approaches in AD, while emphasizing that their effects may depend on disease stage and isoform specificity [50].

Overall, current evidence supports a biologically plausible association between CA II dysregulation and AD, but a direct causal relationship has not yet been established. The finding that CA II inhibition may protect against Aβ toxicity, while CA activation may improve cognitive function, indicates a complex and possibly stage-dependent relationship between CA activity and neurodegeneration. Future studies should be isoform-specific, longitudinal, and clinically controlled before CA I or CA II can be considered validated therapeutic targets or biomarkers in AD.

### 5.6. Obstructive Sleep Apnea

Obstructive sleep apnea (OSA) is characterized by repeated partial or complete obstruction of the upper airway during sleep, leading to intermittent hypoxemia and sleep arousals. Erythrocyte CA I and CA II are the main carbonic anhydrase isoforms involved in CO_2_ transport and bicarbonate buffering in the circulation. Changes in CA activity have also been reported under several physiological stress conditions, including chronic hypoxia, high-altitude adaptation, and intense exercise [6,66].

An important early finding directly linked erythrocyte CA activity with OSA severity. In patients with OSA, CA activity was positively correlated with the apnea–hypopnea index (AHI) and measures of nocturnal hypoxemia. This led to the suggestion that increased CA activity may represent an endotypic feature of OSA and may contribute to ventilatory instability [66]. A later intervention study showed that acetazolamide (AZM) reduced CA activity, AHI, and blood pressure in OSA patients with comorbid hypertension. In contrast, continuous positive airway pressure (CPAP) reduced AHI and blood pressure without changing CA activity. This difference suggests that CA inhibition acts through a pathophysiological mechanism distinct from that targeted by positive airway pressure therapy [42]. The pharmacological relevance of this pathway was further supported by a randomized controlled trial in which AZM reduced both blood pressure and sleep-disordered breathing in hypertensive patients with OSA [43].

More recently, sultiame (STM), a carbonic anhydrase inhibitor with antiepileptic properties, has been investigated as a pharmacological treatment for OSA. In a randomized controlled trial, sultiame was well tolerated and produced consistent, dose-dependent reductions in AHI in patients with moderate-to-severe OSA, with the 200 mg dose showing a favorable risk–benefit profile [47]. The FLOW trial, a multicentre, randomized, double-blind, placebo-controlled, dose-finding phase 2 study, later confirmed that sultiame produced significant dose-dependent improvements in AHI, nocturnal hypoxia, sleep quality, and daytime sleepiness over 15 weeks in 298 adults with moderate-to-severe OSA [20]. These findings represent the strongest clinical evidence so far for a pharmacological CA-targeted approach in OSA. In line with this, a mechanistic study showed that reductions in CA activity in both blood and cerebrospinal fluid were directly associated with improvement in OSA severity, supporting CA activity as both an endotype marker and a pharmacodynamic indicator of treatment response [19].

Overall, the CA isoforms most relevant to OSA are erythrocyte CA I and CA II, together with possible CA-mediated CO_2_ sensing mechanisms in respiratory control centers. Although the exact isoform-specific contributions remain unclear, clinical evidence from AZM and sultiame studies supports CA inhibition as a mechanistically plausible and clinically promising strategy for selected OSA patients, particularly those in whom ventilatory instability is a dominant endotypic feature. Future studies should focus on optimal patient selection, long-term safety, and predictors of response to CA inhibitor therapy [19,20].

### 5.7. Obesity-Related Metabolic Dysfunction

Unlike the mitochondrial isoforms CA VA and CA VB, which provide HCO_3_^−^ as a substrate for acetyl-CoA carboxylase in lipogenesis and for pyruvate carboxylase in gluconeogenesis, CA I and CA II are not directly positioned within adipocyte lipogenic pathways [26,27]. Their evidence base in obesity-related metabolic dysfunction instead comes mainly from human studies linking erythrocyte CA I/II activity, glycosylation, and autoantibody status to insulin resistance and metabolic syndrome, together with a smaller number of adipocyte-level studies on CA II. This evidence is more limited in volume than the mechanistic literature on CA VA, CA VB, and CA III, but it is directly isoform-specific rather than inferential.

Several human studies directly implicate CA I and CA II in obesity-related metabolic dysfunction. Kondo et al. identified a glycosylated form of erythrocyte CA I in patients with diabetes mellitus that showed reduced catalytic and immunological activity compared with the native enzyme, indicating that hyperglycemia can chemically modify CA I and impair its function [67]. Biswas and Kumar reported that erythrocyte CA activity was significantly elevated in patients with insulin resistance and correlated with body mass index, fasting insulin, and methylglyoxal levels, supporting a link between CA activity and the metabolic derangements that accompany obesity [68]. In a study of anti-CA autoantibodies, Alver et al. found that anti-CA I antibodies were significantly more prevalent in subjects with metabolic syndrome than in controls, whereas anti-CA II antibody prevalence did not differ significantly between groups, suggesting that CA I, rather than CA II, may be preferentially exposed to the immune system under metabolic-syndrome-associated oxidative and inflammatory stress [69]. These findings indicate that CA I is measurably altered, both biochemically and immunologically, in human metabolic dysfunction, even though a direct mechanistic role in adipose tissue has not been established.

CA II has more direct, if still preliminary, evidence at the adipocyte level. Lynch et al. showed that CA II protein is expressed in a differentiation-dependent manner in 3T3-L1 and 3T3-F442A adipocytes, appearing only after preadipocytes differentiate into mature adipocytes; unlike CA III, whose concentration fell by 65–90% in the presence of insulin, CA II expression was unaffected by insulin, pointing to a distinct, insulin-independent regulatory pattern for CA II in adipose tissue [70]. Building on this, Ma et al. observed that black tea extract promoted thermogenesis and anti-obesity effects via CA II-mediated activation in adipocytes, with increased CA II expression in brown adipose tissue during thermogenic stimulation [71]. Together, these studies suggest CA II could modulate energy expenditure in adipose tissue, though further research is needed to fully understand its mechanism and physiological importance in human obesity. CA III, another cytosolic isoform, has been identified in adipocytes and hepatocytes in the context of obesity and fatty liver disease. Yamamoto et al. demonstrated that CA III expression rises during pre-obese liver adipogenesis and that inhibiting it reduces hepatic lipid accumulation in rodent models [72]. However, Renner et al.’s work with CA III knockout mice indicated that this isoform is not essential for fatty acid synthesis or for protection against high-fat diet-induced obesity, suggesting a modulatory rather than a fundamental role [73].

For CA I, the evidence regarding its involvement in obesity is limited and largely indirect. While changes in erythrocyte CA I levels might occur secondary to metabolic stress or inflammatory states, CA I has not been identified as a mechanistically significant factor in adipose biology or systemic metabolic dysfunction. Woodman et al. reported that AZM administration in a social stress model increased locomotion and weight loss [74]. This effect, however, most likely resulted from systemic metabolic acidosis due to broad CA inhibition rather than a specific action of CA I.

Among the carbonic anhydrase isoforms implicated in obesity-related metabolic dysfunction, mitochondrial CA VA and CA VB hold the most direct pharmacological relevance due to their provision of HCO_3_^−^ for lipogenic and gluconeogenic carboxylation reactions. CA I shows consistent, disease-associated alterations in human insulin resistance, diabetes, and metabolic syndrome, but current evidence is limited to erythrocyte biochemistry and autoantibody studies rather than a defined adipose-tissue mechanism [67,68,69]. CA II may offer ancillary support through insulin-independent, differentiation-dependent expression and thermogenic regulation in adipose tissue, but the current evidence remains preliminary and primarily preclinical [70,71]. Developing effective anti-obesity therapies targeting CA will necessitate isoform-selective mitochondrial inhibitors and tissue-targeted delivery approaches, which are not yet clinically available.

### 5.8. Cancer: CA I as a Biomarker (Brief Overview)

In cancer biology, membrane-associated isoforms CA IX and CA XII are far more directly implicated in hypoxia-driven extracellular acidification, tumor invasion, metastasis, and therapy resistance than CA I or CA II [23,24,25]. A comprehensive treatment of tumor CA biology is therefore beyond the scope of this review. However, CA I warrants brief mention as a candidate circulating biomarker in selected malignancies.

Wang et al. reported significantly elevated serum CA I levels in patients with stage I non-small cell lung cancer (NSCLC) compared with healthy controls, suggesting potential utility as part of an early-detection biomarker panel [21]. CA I has also been included in multi-marker panels for breast cancer detection, where it demonstrated the highest cancer-to-normal selectivity ratio among the markers evaluated in one study [9]. For CA II, Nordfors et al. detected CA II expression in both tumor cells and endothelial compartments of medulloblastomas and supratentorial primitive neuroectodermal tumors [75], and Annan et al. demonstrated that CA II supports tumor endothelial cell survival under lactic acidosis, with VEGF/VEGFR2 signaling contributing to CA II induction [76]. Hannen et al. reported upregulation of the CA2 gene in temozolomide-resistant glioblastoma stem-like cells, implicating CA II in therapy resistance [77].

These observations support a potential role for CA I as a circulating biomarker and for CA II as a context-dependent modulator of tumor vascular adaptation and chemoresistance. However, these associations are less mechanistically defined than those of CA IX and CA XII in the tumor microenvironment, and their clinical diagnostic or prognostic utility has not been prospectively validated. Future studies should clarify the tumor-specific expression patterns, cellular localization, and clinical significance of CA I and CA II in relation to established tumor CA isoforms. The disease-specific relevance and current evidence level for CA I and CA II are summarized in Table 3.

## 6. Clinical Translation Challenges

Despite growing evidence that CA I and CA II are involved in a wide range of human diseases, several structural and practical barriers still limit the translation of these findings into validated diagnostic or therapeutic applications. These barriers are not specific to carbonic anhydrase biology alone, but they become particularly important because the human CA family contains multiple closely related isoforms, and because CA I and CA II play essential physiological roles in tissues that would inevitably be exposed to systemically administered CA modulators.

### 6.1. Isoform Selectivity

As discussed in Section 4.3, the limited isoform selectivity of most classical CA inhibitors means that agents intended to act on CA II in the ciliary body, renal tubules, or central nervous system routinely co-inhibit CA I and CA II in erythrocytes, and non-classical strategies such as active-site entrance blockers and topical ocular delivery only partially resolve this problem for systemic indications [6,12,17,31,32]. From a clinical-translation standpoint, the practical consequence is that isoform selectivity cannot be treated as a secondary pharmacological refinement: for neurological, metabolic, and vascular indications where systemic dosing is unavoidable, it is the primary determinant of whether a candidate CA I/II-directed therapy will have an acceptable benefit-risk profile [12,17,33].

### 6.2. Systemic Adverse Effects of Broad CA Inhibition

The clinical use of systemic CA inhibitors is limited by adverse effects caused by simultaneous inhibition of CA II in the kidney, brain, and bone, as well as inhibition of CA I and CA II in erythrocytes. Common adverse effects of oral acetazolamide and methazolamide include paresthesia, fatigue, gastrointestinal discomfort, metabolic acidosis, nephrolithiasis, and electrolyte disturbances [6,18]. These effects reflect the essential roles of CA II in renal acid–base regulation, neuronal pH control, and osteoclast function, together with the contribution of erythrocyte CA I and CA II to systemic CO_2_ transport.

This issue is especially relevant for emerging indications such as obstructive sleep apnea and Alzheimer’s disease, where long-term CA modulation may be considered. In such settings, the tolerability of chronic CA inhibition must be carefully evaluated. Dose optimization, intermittent dosing strategies, and tissue-targeted delivery systems, including nanoparticle-based or organ-directed formulations, may help improve the therapeutic index of CA-directed agents [12,17,33].

### 6.3. Biomarker Validation

CA I and CA II have been proposed as disease-associated biomarkers in anemia, atherosclerosis, glaucoma, obstructive sleep apnea, and selected cancers. However, their clinical value as diagnostic or prognostic markers has not yet been confirmed in large, prospective, independently replicated cohorts.

Several issues complicate biomarker development in this area. First, CA I is highly abundant in erythrocytes. Therefore, any condition associated with erythrocyte lysis, increased erythrocyte turnover, or hemolysis may elevate circulating CA I levels, regardless of the underlying disease. This makes it difficult to determine whether increased CA I reflects a specific pathological mechanism or simply erythrocyte release [9,22]. Second, CA II is widely expressed in different tissues, and changes in its circulating or tissue levels may represent secondary responses to inflammation, oxidative stress, tissue remodeling, or pH imbalance rather than a primary disease-driving mechanism. Third, the lack of standardized preanalytical procedures, including sample type, hemolysis control, assay platform, and reference ranges, limits comparability between studies and prevents the establishment of clinically useful thresholds [9,33].

For these reasons, prospective validation studies using large, well-characterized cohorts, standardized assay methods, and clinically meaningful endpoints are needed before CA I or CA II can be recommended as routine biomarkers in any disease context.

### 6.4. Disease-Stage Dependency and Tissue-Specific Consequences

Another important complexity in CA I/II pharmacology is that the effects of CA modulation may vary according to disease stage and tissue context. In Alzheimer’s disease, for instance, both CA inhibition and CA activation have been suggested as possible therapeutic strategies. CA inhibition may be beneficial when Aβ-related mitochondrial injury is dominant, whereas CA activation may be useful when oxidative inactivation of CA I and CA II contribute to impaired pH regulation and synaptic dysfunction [10,45,50]. This indicates that a treatment strategy that is beneficial at one disease stage may be ineffective or even harmful at another.

Similarly, CA II inhibition can be therapeutically useful in the ciliary body for glaucoma, but systemic exposure may simultaneously affect renal acid–base regulation, osteoclast-mediated bone resorption, or cerebrospinal fluid dynamics [6,13]. These examples highlight the need for tissue-targeted delivery systems and disease-stage-specific clinical trial designs in CA I/II-directed pharmacology.

### 6.5. Limited Clinical Evidence for CA Activators

Carbonic anhydrase activators represent an interesting but still clinically underdeveloped pharmacological approach. Preclinical studies suggest potential benefits in neurodegeneration and cognitive dysfunction, but the available evidence is currently limited to in vitro enzyme assays, structural studies, and animal models [10,37,38]. No CA activator has yet entered clinical development.

Important questions remain unresolved, including isoform specificity, blood–brain barrier penetration, in vivo pharmacokinetics, long-term safety, and identification of the patient groups and disease stages most likely to benefit from CA activation. Until these issues are addressed in well-designed translational studies, the clinical relevance of CA activators will remain speculative.

## 7. Conclusions and Future Perspectives

This review evaluated CA I and CA II as biomarkers and therapeutic targets across selected human diseases, with particular emphasis on isoform specificity, strength of evidence, and the distinction between causal mechanisms and secondary disease associations. Overall, the available evidence supports two main conclusions.

First, CA II appears to be the more pharmacologically relevant isoform, with clinical value long established in glaucoma and emerging support in obstructive sleep apnea and epilepsy, and preclinical relevance in Alzheimer’s disease and atherosclerosis. The strength of evidence and the primary supporting studies for each of these associations are summarized in Table 3 and are not repeated here.

Second, CA I currently appears more promising as a disease-associated biomarker than as a direct therapeutic target, with the most consistent signals in atherosclerosis, anemia, and selected malignancies (Table 3). Across these conditions, the main interpretive risk is that elevated CA I may be confounded by erythrocyte lysis, systemic inflammation, or accelerated erythrocyte turnover rather than reflecting a disease-specific mechanism.

Several priorities should guide future research. From a pharmacological perspective, the development of highly selective isoform-specific inhibitors, and ultimately activators, of CA I and CA II individually should be regarded as the single most important unmet need in this field, given how consistently limited isoform selectivity has emerged as the central translational barrier across the diseases discussed in this review. Structural strategies targeting less-conserved regions of the active-site cavity, particularly active-site entrance blockers and external-site binders, may offer promising routes toward improved CA II selectivity over CA I in systemic applications and better selectivity among CA isoforms in neurological and metabolic diseases [12,34,36]. In parallel, tissue-targeted delivery approaches, including topical, intravitreal, and nanoparticle-based formulations, should be further developed to reduce systemic CA I/II inhibition while achieving effective modulation at disease-relevant sites [17,31,33].

From a biomarker perspective, large prospective studies are needed before CA I or CA II can be considered routine diagnostic or prognostic markers. These studies should include well-characterized patient cohorts, standardized assay protocols, hemolysis controls, and clinically meaningful endpoints [9,33]. Attention is needed to determine whether changes in CA I/II reflect a direct role in disease or simply result from erythrocyte release, and to establish disease-specific cut-off values that provide additional information beyond current clinical markers.

Mechanistically, future studies should combine enzyme activity assays, tissue-specific protein expression analysis, oxidative modification assessment, pH imaging, and metabolomics to clarify the causal roles of CA I and CA II in disease. Isoform-specific genetic models, including conditional knockout and overexpression systems in selected tissues, will be especially useful for separating the roles of CA I and CA II from those of other co-expressed CA isoforms. Such studies will provide the mechanistic foundation needed for truly isoform-directed therapeutic strategies.

Beyond these established experimental approaches, emerging computational and multiomic technologies are likely to accelerate isoform-specific CA research. Machine-learning platforms that combine multiple molecular representations with large inhibitor activity datasets have already been used to build isoform-specific classification models and to prioritize candidate CA I/II-selective inhibitors from virtual libraries of millions of compounds prior to experimental testing, illustrating how artificial-intelligence-based drug design can complement the structure-based strategies discussed above in addressing the isoform-selectivity problem [78]. In parallel, single-cell transcriptomics, spatial transcriptomics, and integrated multiomic profiling (transcriptomic, proteomic, and metabolomic) offer a route to mapping CA I and CA II expression, their co-expression with other CA isoforms, and disease-associated regulatory changes at cellular and subcellular resolution within intact tissue architecture. These approaches could substantially extend the bulk-tissue and circulating-biomarker evidence reviewed here and help resolve whether CA I/II alterations are cell-autonomous or reflect the surrounding tissue microenvironment.

In summary, CA I and CA II occupy an important position at the intersection of acid–base physiology, erythrocyte biology, pH-dependent signaling, and disease microenvironment adaptation. This makes them scientifically attractive but also requires careful interpretation. Current translational evidence is strongest for CA II in glaucoma and obstructive sleep apnea, while CA I appears most promising as a context-dependent biomarker in erythrocyte-related and inflammatory conditions. The field now needs to move beyond descriptive associations toward mechanistic precision, with tools that define isoform-specific functions, delivery systems that target the relevant tissues, and clinical endpoints that distinguish true CA-mediated effects from general disease-related changes. Without this level of precision, the gap between biological plausibility and clinical utility will likely remain.

## Figures and Tables

**Table 1:** Comparative biological and translational features of CA I and CA II.

Feature	CA I	CA II	Translational Implication
Enzyme class and localization	Cytosolic α-carbonic anhydrase	Cytosolic α-carbonic anhydrase	Both isoforms are affected by many systemic CA modulators because they share the conserved catalytic framework of human α-CAs [1,3,6].
Main tissue distribution	Predominantly erythrocytes; also reported in colon, kidney, and limited neuronal contexts	Broadly expressed in erythrocytes, kidney, eye, brain, osteoclasts, gastric mucosa, skeletal muscle, liver, and other tissues	CA I is more closely linked to erythrocyte-related processes, whereas CA II has broader organ-level relevance [2,6,9,11].
Relative abundance	Highly abundant in erythrocytes; approximately five- to six-fold higher concentration than CA II	Less abundant than CA I in erythrocytes but widely distributed across tissues	High erythrocyte abundance makes CA I sensitive to hemolysis and erythrocyte turnover as a biomarker [6,9,10,22].
Catalytic efficiency	Lower catalytic efficiency; approximately 15% of CA II activity	Very high catalytic efficiency; among the most efficient known enzymes	CA II is more suitable as a pharmacologically actionable enzyme because of its high catalytic activity and broad physiological relevance [2,6,7,12].
Main physiological roles	CO_2_ transport and intracellular pH buffering in erythrocytes; secondary contribution to renal acid–base handling	Renal bicarbonate reabsorption, urinary acidification, aqueous humor production, cerebrospinal fluid regulation, osteoclast function, neuronal pH regulation	CA II has stronger mechanistic links to clinically targetable physiological processes [6,12,13].
Disease associations	Anemia, atherosclerosis, vascular calcification, inflammatory states, selected cancers	Glaucoma, obstructive sleep apnea, epilepsy, Alzheimer’s disease, renal acid–base disorders, bone-related disorders	CA I is mainly relevant as a disease-associated biomarker; CA II is more relevant as a therapeutic target [9,13,15,17,19,20,21,22].
Biomarker potential	Relatively strong but context-dependent; especially in erythrocyte turnover, inflammation, anemia, atherosclerosis, and selected malignancies	Possible but less specific because of broad tissue distribution	CA I may be useful in biomarker panels, but hemolysis and inflammation must be controlled [9,15,21,22].
Therapeutic target potential	Limited direct clinical application; mostly preclinical evidence in vascular calcification/atherosclerosis	Established in glaucoma; emerging in obstructive sleep apnea; possible relevance in epilepsy and neurodegeneration	CA II is the more clinically validated pharmacological target [6,17,18,19,20,31,32].
Main limitation	Circulating levels may reflect hemolysis, erythrocyte turnover, or systemic inflammation rather than disease-specific mechanisms	Broad tissue distribution increases the risk of systemic adverse effects when inhibited non-selectively	Clinical translation requires isoform selectivity, tissue targeting, and disease-context-specific interpretation [6,9,17,18,33].
Overall translational position	Context-dependent biomarker candidate	Established and emerging therapeutic target	CA I and CA II should not be treated as interchangeable CA isoforms [6,9,12,17].

**Table 2:** Representative carbonic anhydrase modulators relevant to CA I and CA II.

Compound or Class	Main Mechanism	CA I Relevance	CA II Relevance	Clinical or Translational Context	Main Limitation
Acetazolamide	Classical sulfonamide; direct coordination of catalytic Zn^2+^	Inhibits erythrocyte CA I; may contribute to systemic effects	Potent CA II inhibition	Glaucoma, altitude sickness, idiopathic intracranial hypertension, epilepsy; investigated in obstructive sleep apnea [6,17,18,19,42,43]	Poor isoform selectivity; systemic adverse effects such as paresthesia, fatigue, metabolic acidosis, and nephrolithiasis [6,17,18].
Methazolamide	Classical sulfonamide; zinc-binding inhibitor	Inhibits CA I; investigated in vascular calcification/atherosclerosis models	Inhibits CA II; possible relevance in glaucoma and neurodegenerative models	Glaucoma; preclinical atherosclerosis and Alzheimer’s disease-related studies [15,17,18,44,45]	Broad CA inhibition; limited disease-specific selectivity [6,17,18].
Dorzolamide	Topical sulfonamide CA inhibitor	Minimal systemic CA I relevance due to topical use	Local CA II inhibition in ciliary body	Glaucoma [6,18,31,32]	Local ocular adverse effects; limited relevance outside ophthalmology [31,32].
Brinzolamide	Topical sulfonamide CA inhibitor	Minimal systemic CA I relevance due to topical use	Local CA II inhibition in ciliary body	Glaucoma [6,18,31,32]	Local ocular adverse effects; not designed for systemic CA-related diseases [31,32].
Topiramate	Antiepileptic drug with secondary CA-inhibitory activity	Limited or indirect relevance	CA II inhibition may contribute to pH-mediated antiseizure effects	Epilepsy and migraine; possible metabolic effects in some contexts [6,17,18,46]	CA inhibition is not the only mechanism; isoform-specific contribution is difficult to isolate [6,17,46].
Zonisamide	Antiepileptic drug with CA-inhibitory activity	Limited or indirect relevance	CA II inhibition may contribute to antiseizure activity	Epilepsy [6,17,18,46]	Multiple pharmacological mechanisms; CA-specific role remains uncertain [6,17,46].
Sultiame	Sulfonamide-related CA inhibition	May affect erythrocyte CA activity	Relevant to CA-mediated ventilatory control and possibly CA II inhibition	Emerging pharmacological treatment candidate for obstructive sleep apnea; also used in antiepileptic contexts [19,20,47]	Long-term safety and patient selection in obstructive sleep apnea require further clarification [19,20].
Coumarins and sulfocoumarins	Active-site entrance blocking rather than direct Zn^2+^ coordination	Potential for improved selectivity over classical sulfonamides	Potential for isoform-selective CA II modulation	Preclinical drug design [12,34,36]	Limited clinical translation; selectivity and pharmacokinetics require further optimization [12,34,36].
Phenols and polyphenols	Interaction with Zn^2+^-bound water/hydroxide through hydrogen bonding	Possible CA I inhibition depending on scaffold	Possible CA II inhibition depending on scaffold	Experimental pharmacology and scaffold development [12,34]	Usually less clinically developed; potency and selectivity may vary [12,34].
Polyamines	Interaction with the metal-coordinated water/hydroxide system	Experimental relevance	Experimental relevance	Mechanistic and structural studies [12,34]	Limited clinical applicability [12,34].
External-site binders	Interference with proton transfer, including His64-related mechanisms	Potential selectivity advantage	Potential selectivity advantage	Isoform-selective drug development [12,29,34]	Mostly experimental; limited clinical validation [12,34].
Histamine, serotonin, catecholamines	Facilitate proton transfer without blocking the catalytic zinc	Can activate CA isoforms depending on binding context	CA II activation demonstrated in structural/mechanistic studies	Experimental CA activation studies [37,38,39]	Lack of clinical application; poor isoform and tissue specificity [10,37,38].
Amino acids and oligopeptides	Additional proton-shuttling support in the active-site region	Experimental relevance	Experimental relevance, especially for CNS-associated CA studies	Preclinical neurodegeneration and cognitive function research [10,37,38]	Blood–brain barrier penetration, pharmacokinetics, and long-term safety remain unclear [10,37,38].
D-phenylalanine and related activator candidates	Enhancement of proton-transfer step	Possible relevance where CA activity is reduced by oxidative modification	Potential relevance in neurodegenerative models	Preclinical cognitive and Alzheimer’s disease-related studies [10,48,49,50]	No clinical validation; disease-stage-dependent effects possible [10,50].

**Table 3:** Disease-specific relevance and evidence level of CA I and CA II.

Disease/Context	CA I Relevance	CA II Relevance	Current Evidence Level	Main Translational Interpretation
Glaucoma	Indirect relevance; systemic CA I inhibition may contribute to adverse effects of oral CA inhibitors, but CA I is not a primary ocular target [6,17,18,51]	Strong relevance; CA II is a validated pharmacological target for reducing bicarbonate-dependent aqueous humor production and intraocular pressure [6,13,16,31,32]	Strong clinical evidence	CA II is an established therapeutic target in glaucoma, whereas CA I is mainly relevant to systemic off-target effects of non-selective CA inhibition [6,18,31,32].
Atherosclerosis and vascular calcification	Mechanistically implicated in inflammation-driven vascular calcification, VSMC osteogenic reprogramming, macrophage-mediated plaque progression, and potential biomarker/target roles [15,44,54]	Reported in advanced atherosclerotic plaques and osteoclast-like cells, but its pathogenic contribution appears less direct than that of CA I [55,56]	Moderate preclinical evidence; limited clinical validation	CA I appears more directly relevant than CA II in vascular calcification and atherosclerosis, but prospective human studies and isoform-selective tools are needed [15,44,54].
Anemia	Altered erythrocyte CA I patterns have been reported in several anemia subtypes; reduced CA I may help distinguish autoimmune hemolytic anemia and may also be relevant in G6PD-related hemolysis [22,57,58]	CA II may increase in some anemia contexts and may become antigenic under oxidative stress; anti-CA antibodies have also been described in anemia-related conditions [22,58,59,60]	Preliminary to moderate observational evidence	CA I/CA II patterns may reflect erythrocyte pathophysiology, oxidative stress, or immune-mediated processes, but neither isoform is currently validated as a routine diagnostic biomarker [22,58,59,60].
Epilepsy	Mostly indirect relevance, potentially through systemic acid–base regulation, zinc-related mechanisms, or cerebral blood-flow-related effects [6,61]	Relevant to CNS pH regulation and bicarbonate-dependent GABAergic signaling; CA II inhibition may contribute to the effects of several antiepileptic drugs [6,17,46,63]	Moderate mechanistic and pharmacological evidence	CA II is a plausible adjunctive pharmacological target in epilepsy, but its independent contribution must be separated from CA VII and from non-CA mechanisms of antiepileptic drugs [6,17,46,63].
Alzheimer’s disease	May be affected by oxidative modification in AD-related brain regions; possible contribution to impaired pH homeostasis and protein quality control [10,48,49,50]	Implicated in mitochondrial dysfunction, amyloid-β-related toxicity, plaque-associated alterations, and neuronal pH regulation [45,50,64,65]	Preliminary preclinical and observational evidence	CA modulation in AD may be disease-stage dependent; both CA inhibition and CA activation remain experimental and require isoform-specific clinical validation [10,45,50].
Obstructive sleep apnea	Erythrocyte CA I may contribute to systemic CO_2_ transport, bicarbonate buffering, and CA activity changes associated with ventilatory instability [6,42,66]	CA II and overall CA activity are relevant to ventilatory control and pharmacological response to CA inhibitors such as acetazolamide and sultiame [19,20,42,43,47,66]	Emerging clinical evidence	CA inhibition is a promising pharmacological strategy for selected OSA endotypes, particularly those characterized by ventilatory instability, but isoform-specific contributions remain incompletely defined [19,20,42,43,47,66].
Obesity-related metabolic dysfunction	Altered erythrocyte CA I glycosylation and autoantibody status reported in diabetes and metabolic syndrome; no defined adipose-tissue mechanism identified [67,68,69,74]	Differentiation-dependent, insulin-independent expression in adipocytes; possible role in thermogenesis and energy expenditure, but evidence remains preliminary and mainly preclinical [70,71]	Preliminary; mainly preclinical for CA II, mainly human observational for CA I	CA VA and CA VB remain the isoforms most directly linked to lipogenic/gluconeogenic mechanisms; CA I and CA II show isoform-specific human and cellular alterations, respectively, but neither has an established causal role in adipose biology [26,27,67,68,69,70,71,72,73,74].

## Data Availability

No new data were created or analyzed in this study. Data sharing is not applicable to this article.

## Abbreviations

The following abbreviations are used in this manuscript:

Abbreviation	Definition
AD	Alzheimer’s disease
AHI	Apnea–hypopnea index
AI	Artificial intelligence
AZM	Acetazolamide
BAT	Brown adipose tissue
CA	Carbonic anhydrase
CA I	Carbonic anhydrase I
CA II	Carbonic anhydrase II
CA IX	Carbonic anhydrase IX
CA XII	Carbonic anhydrase XII
CAI	Carbonic anhydrase inhibitor
CARP	Carbonic anhydrase-related protein
CNS	Central nervous system
CO_2_	Carbon dioxide
CPAP	Continuous positive airway pressure
GABA	Gamma-aminobutyric acid
HCO_3_^−^	Bicarbonate
HIF	Hypoxia-inducible factor
NSCLC	Non-small cell lung cancer
OSA	Obstructive sleep apnea
ROS	Reactive oxygen species
STM	Sultiame
VSMC	Vascular smooth muscle cell
Zn^2+^	Zinc ion
